# The preoperative prognostic nutritional index is a predictive and prognostic factor of high-grade serous ovarian cancer

**DOI:** 10.1186/s12885-018-4732-8

**Published:** 2018-09-10

**Authors:** Zheng Feng, Hao Wen, Xingzhu Ju, Rui Bi, Xiaojun Chen, Wentao Yang, Xiaohua Wu

**Affiliations:** 1 Department of Gynecological Oncology, Fudan University Shanghai Cancer Center, 270 Dong-an Road, Shanghai, 200032 China; 2 Department of Oncology, Shanghai Medical College, Fudan University, Shanghai, 200032 China; 3 Department of Pathology, Fudan University Shanghai Cancer Center, Shanghai, 200032 China

**Keywords:** Ovarian cancer, Prognostic nutritional index, Residual disease, Platinum sensitivity, Overall survival

## Abstract

**Background:**

The aim of our study was to investigate whether an inflammation-based prognostic score, the prognostic nutritional index (PNI), was associated with clinical characteristics and prognosis in patients with high-grade serous ovarian cancer (HGSC).

**Methods:**

We retrospectively investigated 875 patients who underwent primary staging or debulking surgery for HGSC between April 2005 and June 2013 at our institution. None of these patients received neoadjuvant chemotherapy. Preoperative PNI was calculated as serum albumin (g/L) + 0.005 × lymphocyte count (per mm^3^). The optimal PNI cutoff value for overall survival (OS) was identified using the online tool “Cutoff Finder”. Clinical characteristics and PNI were compared with chi-square or Fisher’s exact tests, as appropriate. The impact of PNI on OS was analyzed using the Kaplan–Meier method and Cox proportional hazards model.

**Results:**

The median (range) PNI was 46.2 (29.2–67.7). The 45.45 cutoff value discriminated patients into the high-PNI and low-PNI groups. A low preoperative PNI was associated with an advanced FIGO stage, increased CA125 level, more extensive ascites, residual disease and platinum resistance. For univariate analyses, a high PNI was associated with increased OS (*p* < 0.001). In multivariate analyses, the PNI remained an independent predictor of OS as a continuous variable (*p* = 0.021) but not as dichotomized groups (*p* = 0.346).

**Conclusion:**

Our study demonstrated that the PNI could be a predictive and prognostic parameter for HGSC.

## Background

Ovarian cancer is one of the most commonly diagnosed and lethal diseases among women worldwide [1]. Although most patients underwent primary surgery and platinum-based adjuvant chemotherapy, half of the patients will relapse within 16 months [2].

Approximately two-thirds of all patients are of advanced stage at diagnosis, with widespread intra-abdominal disease [1, 2]. Patients are at high risk of malnutrition due to cachexia and ascites. Additionally, systemic inflammation also plays an important role during cancer initiation and progression [3]. Accordingly, the identification of relative biomarkers to predict treatment outcomes and prognosis are urgently required.

The prognostic nutritional index (PNI), which can be calculated by the serum albumin concentration and the peripheral blood lymphocyte count, could quantify both the nutritional and immunological status of the body [3, 4]. Currently, the predictive and prognostic role of the PNI has been uncovered in various malignancies [4–9]. However, there are limited data showing the application of the PNI in ovarian cancer [10, 11].

In addition, ovarian cancer is a group of heterogeneous tumors based on distinctive morphological and molecular genetic features [12]. Previous studies have combined these disease subtypes but failed to individually evaluate the clinical and prognostic value of the PNI according to the histologic type.

Since the vast majority of ovarian cancers are high-grade serous ovarian cancer (HGSC), the purpose of our study was to investigate the clinical and prognostic significance of preoperative PNI in HGSC patients.

## Methods

### Clinical data

This study was conducted according to the Declaration of Helsinki and approved by the Committee at Fudan University Shanghai Cancer Center. All participants provided written informed consent.

We retrospectively investigated 875 patients who underwent primary staging or debulking surgery for HGSC between April 2005 and June 2013 at Fudan University Shanghai Cancer Center. The pathological diagnoses were reviewed according to WHO criteria by two experienced gynecologic pathologists.

The inclusion criteria and clinical data collection were consistent with our previous studies [13, 14]. Besides, we also collected BMI, albumin and lymphocyte count data for this study.

Preoperative blood samples from the patient were drawn by antecubital venipuncture within 1 week prior to the operation. The PNI was calculated as serum albumin (g/L) + 0.005 × lymphocyte count (per mm^3^). R0 was defined as the absence of macroscopic residual disease (RD) after surgery. According to the response to platinum-based chemotherapy, patients were clarified as platinum sensitive and platinum resistant [13, 14]. Overall survival (OS) was defined as the time interval from the date of the primary surgery to the date of death or the last follow-up (December 31st, 2016).

### Statistical analyses

SPSS software (version 21.0, SPSS, IBM Inc., Armonk, NY, USA) was used for statistical analyses. Comparisons between categorical variables were performed using chi-square or Fisher’s exact tests, as appropriate. The optimal cutoff value for the PNI was determined via a web-based system Cutoff Finder by Budczies et al. (http://molpath.charite.de/cutoff) [15]. The OS was analyzed with the Kaplan-Meier method and log-rank tests in the univariate analyses. The Cox regression analysis was used for multivariate analyses. *P* < 0.05 was considered statistically significant, and all reported *P* values were 2-sided.

## Results

### Patient characteristics

The patient characteristics are shown in Table 1. The median (range) age of the patients was 56 (30–90) years old. Over 90% of the patients (800/875) had advanced stage (III-IV). After primary surgery, 272 (31.1%) of the patients were debulked to R0 and 603 (68.9%) patients had residual disease. After primary surgery, the majority of patients (849/875, 97.0%) had received platinum-based adjuvant chemotherapy, and 568 (66.9%) patients were platinum sensitive.

**Table 1:** Patient characteristics

Parameters	N	%
Age		
Median (range)	56 (30–90)	
Status		
Died	457	52.20%
Censored	161	18.40%
Alive	257	29.40%
Menopause		
No	273	31.20%
Yes	602	68.80%
FIGO stage		
Early (FIGO I, II)	75	8.60%
Late (FIGO III, IV)	800	91.40%
Family history		
No	643	73.70%
Yes	230	26.30%
BMI		
Median (range)	22.8 (15.6–37.3)	
Underweight (< 18.5)	49	5.70%
Normal (18.5–23.9)	597	69.70%
Overweight (24–27.9)	184	21.50%
Obese (≥ 28)	26	3.00%
PNI		
Median (range)	46.2 (29.2–67.7)	
< 45.45	394	45.50%
≥ 45.45	472	54.50%
CA125		
<500	193	22.60%
≥ 500	662	77.40%
Ascites		
No	99	11.30%
<500 ml	146	16.70%
≥ 500 ml	629	72.00%
Residual disease		
No	272	31.10%
Yes	603	68.90%
Platinum sensitivity		
NA	44	5.20%
Yes	568	66.90%
No	237	27.90%

### Cutoff point for determining the PNI

**Fig. 1:**
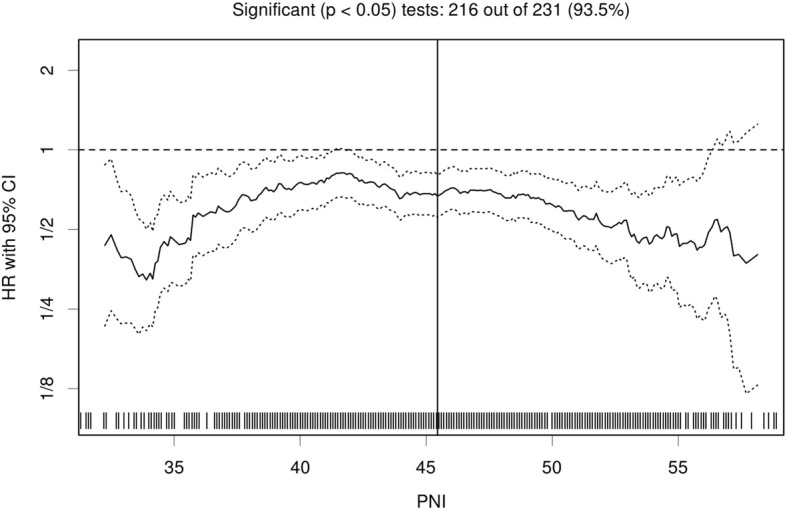
Hazard ratio (HR) for overall survival (OS), independent of the cutoff point for the prognostic nutritional index (PNI), in patients with high-grade serous ovarian cancer (HGSC). The vertical line denotes the optimal cutoff point, which was generated by Cutoff Finder

The PNI ranged from 29.2 to 67.7, with a median level of 46.2. Based on the Cutoff Finder tool, a wide range of cutoff points for the PNI was significant with respect to OS (Fig. 1). In addition, the optimal cutoff point of the PNI was 45.45. Then, patients were divided into high PNI (PNI ≥ 45.45, *n* = 472, 54.5%) and low PNI groups (PNI <  45.45, *n* = 394, 45.5%).

### Correlations between the PNI and clinical characteristics

Correlations of the clinical characteristics with the preoperative PNI are summarized in Table 2. A low preoperative PNI level was associated with an advanced FIGO stage (*p* < 0.001), an increased CA125 level (*p* < 0.001) and more extensive ascites (p < 0.001). Patients who presented with a high PNI level tended to have no residual disease (*p* < 0.001). A greater proportion of the patients in the high PNI group were platinum sensitive compared with those in the low PNI group (76.6% vs 62.8%, *p* < 0.001). We found that underweight patients and obese patients tended to have low PNI levels compared with the others (*p* = 0.023).

**Table 2:** Correlation of PNI and HGSC patient characteristics (*n* = 875)

Parameters	N (%)	PNI		P value
		< 45.45 N (%)	≥ 45.45 N (%)	
Age				
< 56	423 (48.8%)	181 (45.9%)	242 (51.3%)	0.133
≥ 56	443 (51.2%)	213 (54.1%)	230 (48.7%)	
FIGO stage				
Early (I, II)	75 (8.7%)	18 (4.6%)	57 (12.1%)	< 0.001
Advanced (III, IV)	791 (91.3%)	376 (95.4%)	415 (87.9%)	
CA125				
< 500	190 (22.5%)	65 (16.9%)	125 (27.1%)	< 0.001
≥ 500	656 (77.5%)	319 (83.1%)	337 (72.9%)	
Ascites				
No	99 (11.4%)	14 (3.6%)	85 (18.0%)	< 0.001
< 500	144 (16.6%)	27 (6.9%)	117 (24.8%)	
≥ 500	622 (71.9%)	353 (89.6%)	269 (57.1%)	
Residual disease				
No	270 (31.2%)	77 (19.5%)	193 (40.9%)	< 0.001
Yes	596 (68.8%)	317 (80.5%)	279 (59.1%)	
Platinum sensitivity				
Yes	561 (70.5%)	223 (62.8%)	338 (76.6%)	< 0.001
No	235 (29.5%)	132 (37.2%)	103 (23.4%)	
BMI				
Underweight (< 18.5)	47 (5.5%)	27 (7.0%)	20 (4.3%)	0.023
Normal (18.5–23.9)	591 (69.8%)	250 (64.8%)	341 (74.0%)	
Overweight (24–27.9)	184 (21.7%)	94 (24.4%)	90 (19.5%)	
Obese (≥ 28)	25 (3.0%)	15 (3.9%)	10 (2.2%)	

### Prognostic impact of the PNI

The median follow-up time was 41 (1–134) months. A total of 257 (29.4%) patients were still alive at last follow-up, and 457 (52.2%) deaths were documented. The median (95% CI) OS was 55 (49.3–60.7) months.

The known negative influences of an advanced FIGO stage (< 0.001), the presence of residual disease (p < 0.001), and platinum resistance (p < 0.001) for OS were confirmed by univariate analyses.

**Fig. 2:**
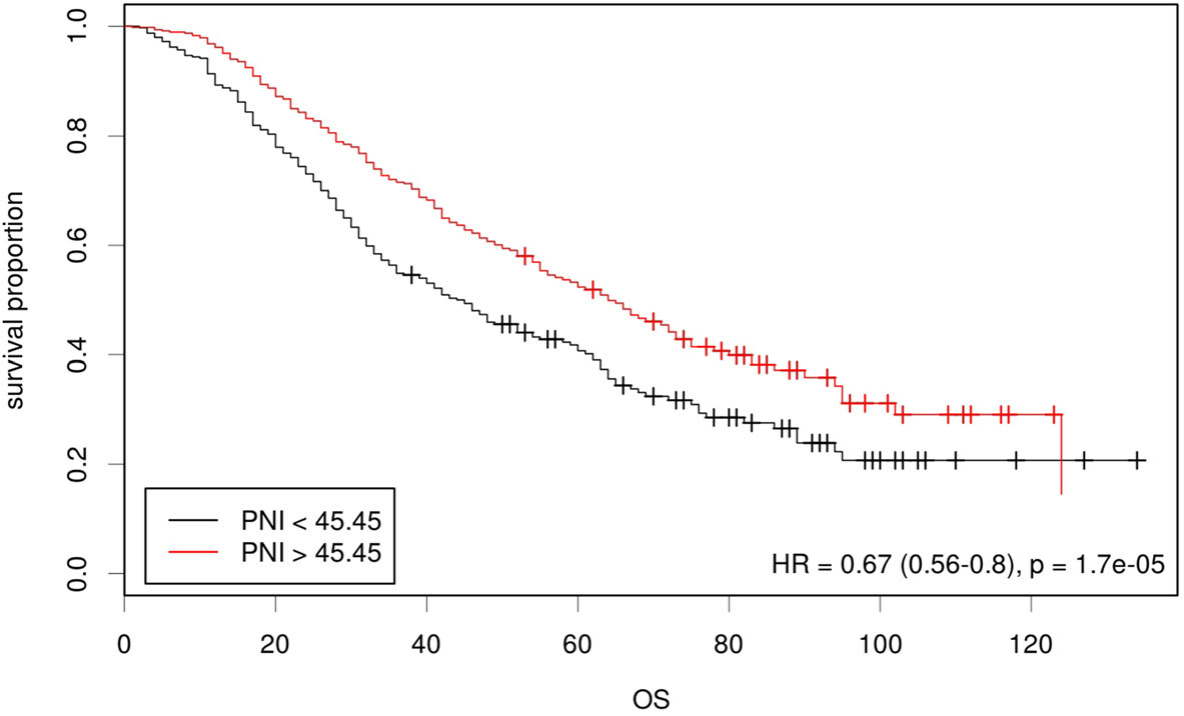
Prognostic value of the prognostic nutritional index (PNI) for overall survival (OS). Kaplan-Meier curve with the log-rank test

In the univariate analysis, a high PNI was associated with prolonged OS (64.0 (55.8–72.2) vs 44.0 (36.8–51.2) months, *p*<0.001, Fig. 2). In the multivariate analysis with adjustments for age, FIGO stage, residual disease and platinum sensitivity status, the PNI was also an independent predictor for OS as a continuous variable (HR = 0.980, 95% CI, 0.964–0.997, *p* = 0.021; Table 3). However, the PNI was not independently associated with OS as dichotomized groups (HR = 0.907, 0.741–1.111, *p* = 0.346; Table 4).

**Table 3:** Cox regression of OS using PNI as a continuous variable

Characteristics	OS		
	HR	95%CI	P value
Age (continuous variable)	1.013	1.002–1.024	0.023
FIGO stage			
Early (I, II)	Referent		
Advanced (III, IV)	2.761	1.597–4.774	< 0.001
Platinum sensitivity			
No	Referent		
Yes	0.205	0.166–0.253	< 0.001
Residual disease			
Yes	Referent		
No	0.765	0.593–0.988	0.040
PNI (continuous variable)	0.980	0.964–0.997	0.021

**Table 4:** Cox regression of OS using PNI (< 45.45 vs ≥ 45.45) in multivariate analysis

Characteristics	OS		
	HR	95%CI	P value
Age (continuous variable)	1.012	1.001–1.023	0.027
FIGO stage			
Early (I, II)	Referent		
Advanced (III, IV)	2.839	1.643–4.904	< 0.001
Platinum sensitivity			
No	Referent		
Yes	0.207	0.168–0.255	< 0.001
Residual disease			
Yes	Referent		
No	0.734	0.569–0.946	0.017
PNI			
< 45.45	Referent		
≥ 45.45	0.907	0.741–1.111	0.346

## Discussion

In this large single institutional study with ten-year follow-up, we demonstrated that the preoperative PNI was associated with clinical characteristics and treatment outcomes. A low PNI level was correlated with impaired OS in Chinese patients with HGSC.

The immunological and nutritional condition could undoubtedly influence patient outcomes, and various relative markers have been established [16–22]. Among these markers, the PNI could reflect both the nutritional and immunological statuses of the host and has been validated as an indicator for treatment outcomes in various malignancies [4–9].

The data on ovarian cancer are quite limited. Miao et al. [10] reported that the PNI could help predict the platinum resistance of ovarian cancer (AUC = 0.688), and in their cohort, the PNI was also an independent prognostic factor for PFS and OS. Zhang et al. [11] further compared several inflammation- and nutrition-related factors and found that the PNI was superior to C-reactive protein/albumin ratio (CAR), modified Glasgow prognostic score (mGPS), and lymphocyte/monocyte ratio (LMR).

These findings are consistent with previously published data. We observed that low PNI levels were associated with an advanced FIGO stage, an increased CA125 level and more extensive ascites. Thus, higher tumor burden could likely lead to malnutrition and immune suppression, potentially reflected by a low PNI level. Thus, the PNI could reflect tumor burden and was undoubtedly correlated with debulking outcomes for ovarian cancer patients.

In addition, the chemotherapeutic response could be influenced by residual tumor, patient tolerability, and host defense against cancer. Consistent with Miao et al. [10], we propose that a greater proportion of the patients in the high PNI group were platinum sensitive compared with those in the low PNI group. Therefore, the comprehensive nutritional and immune indicator could help predict ovarian cancer patient platinum sensitivity.

Furthermore, both Miao et al. [10] and Zhang et al. [11] reported that the PNI was an independent prognostic factor. However, in the present cohort, the PNI was also an independent predictor for OS as a continuous variable but not as dichotomized groups. The underlying reason was that the definitive normal range of the PNI has not been determined, and the border value to dichotomize the study population into high and low PNI groups is difficult to determine. An epidemiological survey is required for the generation of a normal range.

A notable limitation is that the present study was a retrospective study with potential recall bias. However, the completeness of clinical data, up to 10-year follow-up duration and the large sample size could partly compensate for this limitation. Notwithstanding its limitation, this study was a single institutional retrospective study involving a group of homogeneous patients with the same histology who underwent similar treatment strategies, and the PNI is available in routine blood tests and can be popularized in clinical application.

## Conclusion

Our study indicated that preoperative PNI could reflect tumor burden and thus indicate clinical outcomes to a certain extent. This index should be regarded as a predictive and prognostic factor for our HGSC patients.

## Abbreviations

HGSC: High-grade serous ovarian cancer (LGSC)
OS: Overall survival; RD, Residual disease
PNI: Prognostic nutritional index
